# Plague and Sanitation

**Published:** 1905-05-27

**Authors:** 


### The Hospital.

A JOURNAL OF

The Medical Sciences and Hospital Administration.

VOL. XXXVIII.—No. 974. SATURDAY, MAY 27, 1905.

### PLAGUE AND SANITATION.

THE elaborate paper on Plague in India, recently read before the Indian Section of the Society of Arts by Dr. Creighton, afforded a very curious example of the tendency to remain standing in ancient ways which has from time to time been displayed by men of acute and penetrating intellect. Dr. Creighton has recently visited India for the purpose of personally examining into the conditions in which plague has found such abundant opportunities for development and increase; and he informs us, as might have been expected, that these are such as have been known, now for a generation, as filth conditions, and as being generally favourable to epidemic diseases of every description. The histories of plague which may be found, more or less condensed, in any medical book of reference, in "Quain's Dictionary," for example, would have told him as much as this without the trouble of going to find it out; and would indeed have placed before him, in the descriptions of plague-infected localities in China and in Chinese Tartary, accounts of filth before which those which he related to the Society would be compelled to pale their ineffectual fires. It is obvious that the mere superabundance of filth, while it marks out a locality as one likely to be invaded by disease, and as certain to suffer severely whenever the invasion may occur, throws no light at all upon the probable character of the invader, and does not enable us to predict, with any approach to certainty, whether plague, or typhus, or cholera will be the particular scourge by which Nature will strive to call the attention of man to the error of his ways. This issue will depend, in all probability, upon the presence or absence of sources of one or of another infection, and hence, in the long run, upon the diseases which may be current in other localities between which and the filthy locality there may at the time be frequent intercourse. It has been one of the most important achievements of modern sanitary science to ascertain that, although the active causes of many infectious diseases are assisted in their operation by the presence of filth, yet that this filth is but a condition favourable to the growth and to the continued vitality of some specific contagion, which, in the case of many diseases, has been identified as a recognisable bacillus, and which on good grounds is suspected to be of this nature in all cases. Dr. Creighton, to all appearance, stops short of the adoption of this conclusion; and, instead of recognising in filth merely a condition favourable to the vitality of saprophytic bacilli, he appears to regard it as one which is dangerous in its own nature, and which should itself be looked upon as an active cause of a specific contagious malady. He casts doubt upon the value of the work done by bacteriologists, and asks for that of "epidemiologists" instead. His mental attitude, in fact, is a little suggestive of one who, in these days of railroads, should decline to travel by their agency, and should stay at home because there were no stage coaches upon the road to what would otherwise have been his destination.

We need hardly remind our readers that one of the most important consequences of the tracing of distinct contagions to specific bacilli has been the acquisition of a power of conferring immunity against these contagions by the employment of the very toxins which the bacilli themselves produce; a power, in other words, of referring the protection afforded by vaccinia to a far-reaching general principle, and of applying this principle to a great variety of diseases. Not the least important of its applications has been to plague; and, although the success obtained has by no means been unchequered, although accidents have happened which might perhaps have been avoided by more careful management, and although there is evidently much to be learnt in connection with the subject, it is none the less manifest, to all who have come to the study of the facts with unbiassed minds, that inoculation has already been of immense service in India, and that it has preserved the populations of many localities from what would in all probability have been extensive and fatal outbreaks of the disease. Dr. Creighton has nothing to say about it except to record the refusal of some particular community to submit themselves to the process; and he turns away from modern science and its applications, in order to declare the cause of plague to be an earth poison, which, if we understand him correctly, he believes to be of spontaneous origin in soils which have become saturated with filth in excess of their power to dispose of it by oxidation or other chemical change, to be drawn into the interior of houses during the continuance of the comparatively lower temperature of night, and to be, we presume, of a gaseous or vaporous character. He explains its rise and fall in the subsoil by the changes in the level of subsoil water to which Pettenkofer, many years ago, called attention in relation to epidemics of cholera; and he explains the mortality among rats by the circumstance that they are subterranean dwellers, and therefore are among the first to be exposed to the action of the poison. Dr. Creighton points to no remedy but the complete reconstruction of the Indian villages in the plains, and the substitution, for the habitations which he describes, and which are built of mud excavated from the soil of the site, and have the walls and the floors alike permeable by gaseous emanations, of buildings with sound foundations and floors, and with damp-proof courses in the walls. On various grounds the question might perhaps be shown to be a less simple one than Dr. Creighton appears to consider it; and it is manifest that his remedy, the complete reconstruction of village habitations, can hardly be regarded in any other light than as a counsel of unattainable perfection.

